# Comparison of Pressure Changes by Head and Neck Position between High-Volume Low-Pressure and Taper-Shaped Cuffs: A Randomized Controlled Trial

**DOI:** 10.1155/2015/386080

**Published:** 2015-10-05

**Authors:** Nobuyasu Komasawa, Ryosuke Mihara, Kentaro Imagawa, Kazuo Hattori, Toshiaki Minami

**Affiliations:** Department of Anesthesiology, Osaka Medical College, Daigaku-machi 2-7, Takatsuki, Osaka 569-8686, Japan

## Abstract

The present study compared changes in cuff pressure by head and neck position between high-volume low-pressure (HVLP) and taper-shaped (taper) cuffs in a prospective randomized clinical trial.* Methods*. Forty patients were intubated using tracheal tubes with either HVLP (*n* = 20; HVLP group) or taper-shaped (*n* = 20; Taper group) cuffs. Initial cuff pressure was adjusted to 15, 20, or 25 cmH_2_O in the neutral position. Cuff pressure was evaluated after changing the head and neck positions to flexion, extension, and rotation.* Results*. Cuff pressure significantly increased with flexion in both HVLP and Taper groups at all initial cuff pressures. It significantly increased with extension in the HVLP group, but not in the Taper group. Cuff pressure did not significantly differ with rotation in either group and was significantly smaller in the Taper group during flexion and extension than in the HVLP group, regardless of initial cuff pressure.* Conclusion*. Cuff pressure changes with head and neck flexion and extension were smaller in the Taper group than in the HVLP group. Our results highlight the potential for taper cuffs to prevent excessive cuff pressure increases with positional changes in the head and neck. This trial is registered with UMIN000016119.

## 1. Introduction

The safety margin of tracheal tube cuff pressure lies between excessive and insufficient pressure. Specifically, insufficient pressure can lead to air leakage, which lessens the effect of mechanical ventilation and results in the leakage of inhalation anesthetics [1], while excessive pressure can cause serious injury and affect blood flow to the tracheal mucosa, resulting in tracheal stenosis, fistula, or tracheal rupture [2, 3]. Movement of the head and neck and the resulting displacement of the tracheal tube can alter cuff pressure [4].

Cuff shape design has advanced substantially in recent years. For example, the taper-shaped cuff was developed with a cylindrical shape to seal the trachea better than existing cuffs such as the high-volume low-pressure (HVLP) cuff [5, 6].

We hypothesized that the taper-shaped cuff would prevent cuff pressure changes due to head and neck position changes more effectively than the HVLP cuff. To this end, we compared cuff pressure increases with different head and neck positions between the HVLP and taper-shaped cuffs in a prospective randomized controlled trial.

## 2. Methods

**Figure 1:**
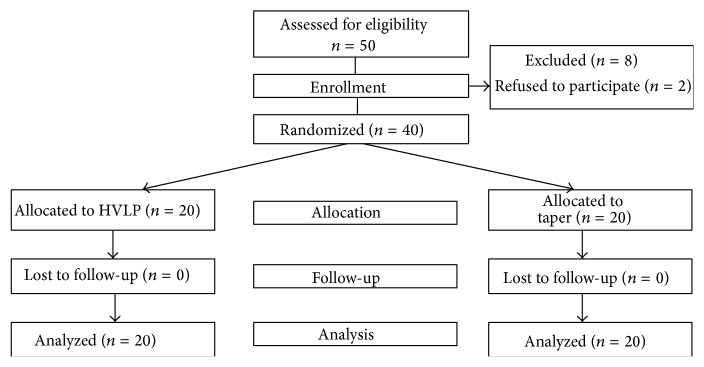
CONSORT flowchart for patient recruitment.

The Research Ethics Committee of Osaka Medical College approved this study.  Figure 1 shows the CONSORT flowchart for participant recruitment. This study was registered in the UMIN Clinical Trials Registry (registration number: UMIN000016119). From January to April 2015, eligibility was assessed for 50 patients, of whom two refused and eight were excluded in accordance with the eligibility criteria. After obtaining written informed consent, 40 patients aged 20 to 75 years who were to undergo general anesthesia in a supine position were randomly assigned (envelope method) to one of two groups: intubation by a tracheal tube with the HVLP cuff (HVLP group, 20 patients) or the taper-shaped cuff (Taper group, 20 patients). Exclusion criteria were morbid obesity defined by a body mass index >35, cervical disease or cervical movement restriction, gastroesophageal reflux, previous upper abdominal surgery, and a recent (within seven days) history of upper respiratory tract infection [7].

**Figure 2:**
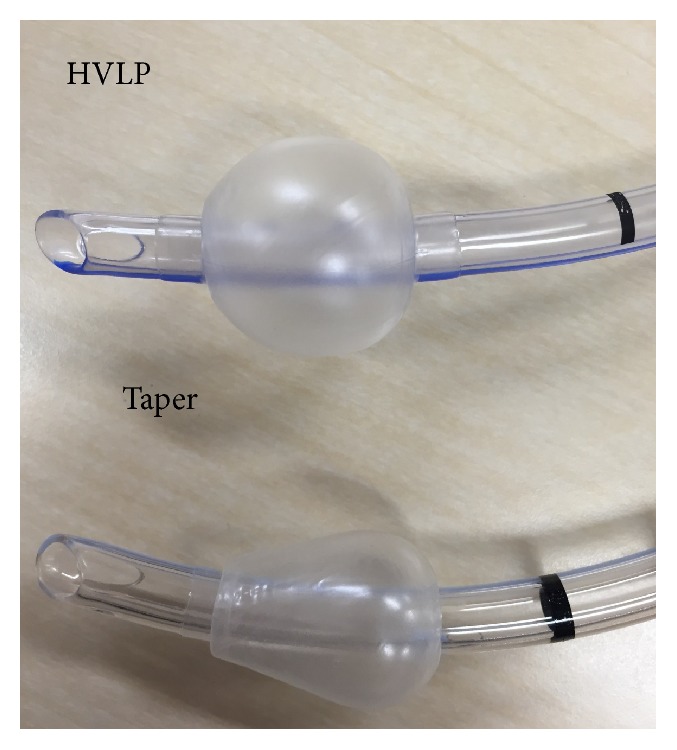
Shapes of high-volume low-pressure (HVLP) and taper-shaped (taper) cuffs.

Percutaneous oxygen saturation, noninvasive blood pressure, heart rate, electrocardiography, and end-tidal carbon dioxide tension were monitored for each patient [7]. Without any premedication, anesthesia was induced with a bolus infusion of propofol 1-2 mg·kg^−1^ and remifentanil 0.3–0.5 *μ*g·kg^−1^·min^−1^. Rocuronium 0.8–1.0 mg·kg^−1^ was administered as a muscle relaxant. Anesthesia maintenance was performed with continuous inhalation of sevoflurane, and no nitrous oxide was used. A McL blade of either size 3 or size 4 was used according to the anesthesiologist's preference. Anesthesiologists performed intubation using a tracheal tube with a HVLP cuff (Portex Soft Seal, Smith Medical Co., Ltd., Kent, UK) or taper-shaped cuff (Mallinckrodt TaperGuard, Covidien, Dublin, Ireland) according to the randomization. The shapes of the cuffs are shown in Figure 2. The size of the tracheal tube was determined by the anesthesiologist based on the formula of height/20 mm to standardize study conditions. The number of intubation trials and Cormack's classification were assessed. The tip of the tracheal tube was placed about 2 cm into the trachea and fixed at the center of the mouth with cohesive tape Durapore [8]. Mechanical ventilation was performed in a volume-controlled manner at a rate of 10–12 mL/kg and 8–10 times/min.

Cuff pressure adjustments and measurements were performed with an automated cuff pressure controller (Mallinckrodt Pressure Control, Covidien, Dublin, Ireland), which is accurate to 1 decimal place, according to the manufacturer. The cuffs were initially inflated to a pressure of 15, 20, or 25 cmH_2_O. The head and neck were placed in the neutral position, such that the external ear canal is level with the top of the shoulder and the ear-eye line (from the external ear canal to the superior orbital margin) is vertical, and then repositioned randomly in the following positions: maximal extension, maximal flexion (about 45 degrees), or maximal rotation (about 70–90 degrees) to the right. Cuff pressure was recorded by an independent observer in each position. We measured cuff pressure changes at the expiratory pauses for roughly 30 seconds at each position. After each cuff pressure measurement, the participant was returned to a neutral position. All participants experienced three initial cuff pressures for neutral, flexion, extension, and rotation, for a total of 12 patterns [9].

Statistical analysis was performed using JMP 11 (SAS Institute Inc., Cary, NC, USA). A two-way repeated measures analysis of variance was used to compare cuff pressure changes. Data are presented as either mean ± standard deviation or median ± interquartile range. *P* < 0.05 was considered statistically significant.

Sample size calculation was based on data from five participants in a pilot study in which cuff pressures were measured in the four flexion positions. The largest difference in mean oropharyngeal leak pressure between the positions was 4 ± 4.9 cmH_2_O. Eighteen patients were needed to detect a difference in oropharyngeal leak pressures between positions with a type I error of 0.05 and a power of 0.8. Hence, 20 patients were enrolled in this study in order to allow for any methodological difficulties that could lead to exclusion from the study.

## 3. Results

Patient characteristics are summarized in Table 1. None of the patients were lost to follow-up during the trial.

### 3.1. Cuff Pressure Changes according to Changes in Head and Neck Positions for the HVLP and Taper Groups

Table 2 shows the cuff pressure changes according to initial cuff pressures. During flexion and extension, cuff pressure changes were significantly higher in the HVLP group than in the Taper group, regardless of initial cuff pressure (*P* < 0.001 for both flexion and extension). In contrast, cuff pressure changes did not differ significantly between the two cuff types during rotation, regardless of initial cuff pressure.

### 3.2. Cuff Pressure as Affected by Head and Neck Position in the HVLP and Taper Groups

**Figure 3:**
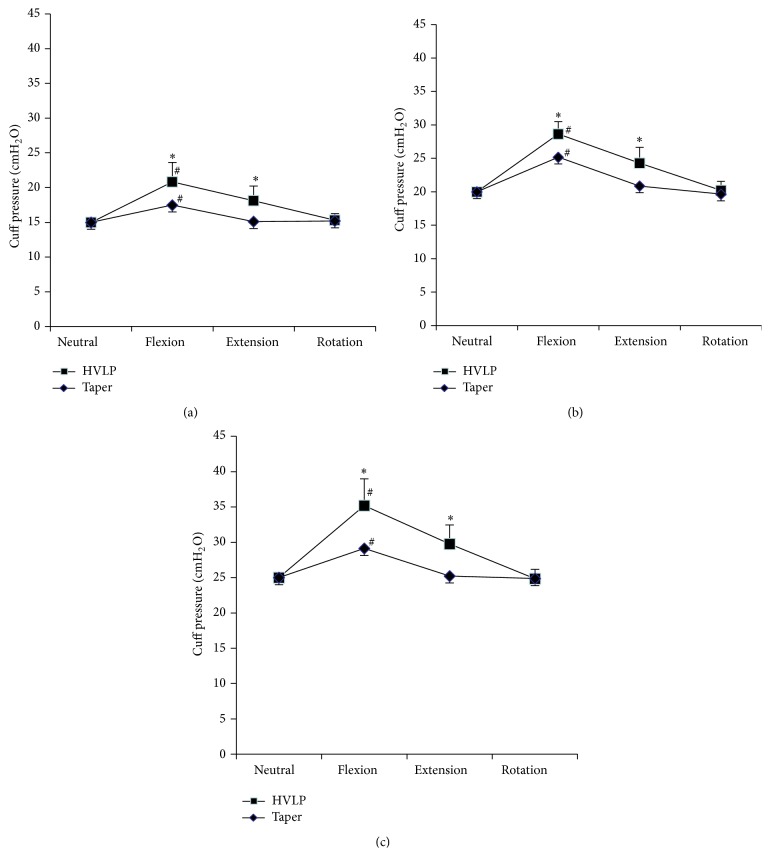
Cuff pressure by head and neck position with HVLP and taper cuffs. HVLP group: trachea was secured using a tracheal tube with a high-volume low-pressure cuff; Taper group: trachea was secured using a tracheal tube with a taper-shaped cuff. ^*∗*^ *P* < 0.05 compared to taper. ^#^ *P* < 0.05 compared to neutral position. (a) Initial cuff pressure of 15 cmH_2_O, (b) initial cuff pressure of 20 cmH_2_O, and (c) initial cuff pressure of 25 cmH_2_O.

Figure 3 shows changes in cuff pressure by head and neck position at the three initial cuff pressures. Cuff pressure significantly increased with flexion in both HVLP and Taper groups at initial cuff pressures of 15, 20, and 25 cmH_2_O. Cuff pressure significantly increased with extension in the HVLP group, but not in the Taper group, regardless of initial cuff pressure. Cuff pressure did not significantly change with rotation in both HVLP and Taper groups. Cuff pressure in the Taper group was significantly smaller during flexion and extension than in the HVLP group, regardless of initial cuff pressure.

### 3.3. Number of Patients for Whom Cuff Pressure Exceeded 30 cmH_2_O

At an initial cuff pressure of 20 cmH_2_O, cuff pressure exceeded 30 cmH_2_O for 5 patients in the HVLP group; this was not observed for any patients in the Taper group (*P* < 0.001). At an initial cuff pressure of 25 cmH_2_O, cuff pressure exceeded 30 cmH_2_O in 18 patients in the HVLP group and in 6 patients in the Taper group during flexion (*P* < 0.001). During extension, this was also observed in 10 patients in the HVLP group, but not in any patients in the Taper group (*P* < 0.001).

## 4. Discussion

Tracheal intubation during general anesthesia is essential for procedures that require changes in head and neck position. For example, sufficient extension of the neck is needed for thyroid or otolaryngology surgery, while neck flexion is often required for cervical spine, plastic, and cerebral surgery. Since head and neck movement changes the shape of the pharynx or compresses the trachea, cuff pressure management is important for preventing tracheal stenosis, necrosis, and postoperative pharyngeal pain [10, 11].

Introduction of a cuffed endotracheal tube with improved tracheal sealing characteristics over existing cuffed tubes may encourage the regular use of these tubes in the clinical setting. The pressure exerted by HVLP cuffs, popularized in the 1970s, is almost equal to the cuff inflation pressure. HVLP cuffs can be used for positive pressure ventilation with a cuff inflation pressure of less than 30 cmH_2_O [12]. In this study, cuff pressure exceeded 30 cmH_2_O in roughly 90% of patients during flexion and in 50% during extension, at an initial cuff pressure of 25 cmH_2_O, demonstrating the potential inherent risk of increased cuff pressure leading to tracheal wall ischemia by using HVLP cuffs.

The taper, a newly developed endotracheal tube, has a cylindrical cuff that seals the trachea better than existing endotracheal tubes. This tube is shaped for effective prevention of leakage via longitudinal folds. Previous studies have assessed the sealing ability of taper-shaped cuffs using water or viscous fluids in adult airway simulation models [13, 14]. Our study revealed that the taper had significantly smaller cuff pressure increases during flexion or extension relative to those observed when using the HVLP. Moreover, cuff pressure exceeded 30 cmH_2_O in only 30% of patients during flexion.

We found that cuff pressure with flexion was significantly smaller in the Taper group compared to the HVLP group, regardless of initial cuff pressure, and that cuff pressure increased with flexion and extension in the HVLP group. In the Taper group, cuff pressure increased significantly with flexion but did not increase with extension or rotation. The cuff pressure was also significantly smaller in the Taper group than in the HVLP group with flexion and extension at all initial cuff pressures. We surmise that the taper prevents excessive cuff pressure increases when changes occur in head and neck positioning. One probable reason for the differences observed between the taper-shaped and HVLP cuffs may be differences in the cuff attaching area [15]. Since the area of the cuff that attaches to the tracheal wall is smaller with taper-shaped cuffs than with HVLP cuffs, the attachment pressure of taper-shaped cuffs may be higher than with HVLP cuffs under the same cuff pressure. Given these advantages and the cylindrical shape, taper cuffs are expected to reduce the incidence of excessive cuff pressure increases.

Notably, we did not observe any cuff pressure increases with rotation in either the HVLP or the taper, which differed from other study findings [4]. This inconsistency may be partially due to anatomical differences between children and adults. While the tracheal tube may rotate smoothly in the trachea and not elicit pressure changes in adults, pediatric tracheal tube cuffs can become compressed even by head rotation.

The present study has a number of limitations. Data were collected by unblinded observers. Blinding was unrealistic in this study because the position of the head and neck was difficult to hide from the observer who recorded the data [16]. However, the measured variables in this study were clearly defined. Thus, the lack of blinding is unlikely to have skewed our results. Second, as this study was conducted at a single institute, a large-scale multicenter study or meta-analysis will be needed to clarify the utility of taper-shaped cuffs for use in situations involving changes in head and neck position [17].

In conclusion, we demonstrated that changes in cuff pressure with head and neck flexion and extension were smaller in the Taper group than in the HVLP group. Our results highlight the potential for taper cuffs to help prevent excessive cuff pressure increases upon changes in head and neck position.

## Figures and Tables

**Table 1:** Patient characteristics of each group presented as mean ± SD or number of patients. HVLP group: trachea was secured with a high-volume and low-pressure cuffed tracheal tube; Taper group: trachea was secured with taper cuffed tracheal tube.

	HVLP group *N* = 20	Taper group *N* = 20
Age (years)	61.8 ± 12.9	64.9 ± 12.7
Gender (male/female)	10/10	10/10
Body weight (kg)	59.4 ± 13.7	57.3 ± 7.9
Height (cm)	161.6 ± 7.5	159.5 ± 9.9
BMI (kg/m^2^)	22.6 ± 4.6	22.5 ± 2.5
Mallampati score (1/2/3/4)	8/11/1/0	7/12/1/0
Cormack-Lehane grade (1/2/3/4)	11/8/1/0	9/11/0/0
Tracheal tube size (7.0/7.5/8.0/8.5)	2/11/6/1	3/7/8/2

BMI: body mass index.

ASA: American Society of Anesthesiologists.

**Table:** (a) Initial cuff pressure 15 cmH_2_O

	Flexion	Extension	Rotation
HVLP	5.3 [3.9–7.7]	3.4 [2.4–4.2]	0.3 [−0.8–1.1]
Taper	2.6 [1.5–3.5]	0.2 [−0.2–0.4]	0.1 [−0.7–0.6]
*P* value	<0.001^*∗*^	<0.001^*∗*^	0.41

**Table:** (b) Initial cuff pressure 20 cmH_2_O

	Flexion	Extension	Rotation
HVLP	8.4 [7.6–9.4]	4.7 [3.9–5.4]	0.4 [−0.4–1.2]
Taper	5.4 [4.0–6.5]	0.9 [0.4–1.8]	−0.6 [−1.5–0.4]
*P* value	<0.001^*∗*^	<0.001^*∗*^	0.12

**Table:** (c) Initial cuff pressure 25 cmH_2_O

	Flexion	Extension	Rotation
HVLP	10.3 [8.5–13.1]	5.0 [2.7–7.0]	−0.1 [−0.4–0.5]
Taper	4 [2.6–5.3]	0.5 [−0.4–1.4]	0.1 [−1.0–0.9]
*P* value	<0.001^*∗*^	<0.001^*∗*^	0.91

## References

[1] El-Orbany M., Salem M. R. (2013). Endotracheal tube cuff leaks: causes, consequences, and management. *Anesthesia and Analgesia*.

[2] Kim D., Jeon B., Son J.-S., Lee J.-R., Ko S., Lim H. (2015). The changes of endotracheal tube cuff pressure by the position changes from supine to prone and the flexion and extension of head. *Korean Journal of Anesthesiology*.

[3] Dobrin P., Canfield T. (1977). Cuffed endotracheal tubes: mucosal pressures and tracheal wall blood flow. *The American Journal of Surgery*.

[4] Kako H., Krishna S. G., Ramesh A. S. (2014). The relationship between head and neck position and endotracheal tube intracuff pressure in the pediatric population. *Paediatric Anaesthesia*.

[5] Shiotsuka J., Lefor A. T., Sanui M., Nagata O., Horiguchi A., Sasabuchi Y. (2012). A quantitative evaluation of fluid leakage around a polyvinyl chloride tapered endotracheal tube cuff using an in-vitro model. *HSR Proceedings in Intensive Care & Cardiovascular Anesthesia*.

[6] Komasawa N., Fujiwara S., Miyazaki S., Soen M., Minami T. (2014). Comparison of fluid leakage from four different cuffed pediatric endotracheal tubes using a pediatric airway simulation model. *Pediatrics International*.

[7] Miyazaki Y., Komasawa N., Matsunami S., Kusaka Y., Minami T. (2015). Laryngoscopy facilitates successful i-gel insertion by novice doctors: a prospective randomized controlled trial. *Journal of Anesthesia*.

[8] Komasawa N., Fujiwara S., Miyazaki S., Ohchi F., Minami T. (2015). Shifts in endotracheal tube position due to chest compressions: a simulation comparison by fixation method. *Journal of Emergency Medicine*.

[9] Sanuki T., Uda R., Sugioka S. (2011). The influence of head and neck position on ventilation with the i-gel airway in paralysed, anaesthetised patients. *European Journal of Anaesthesiology*.

[10] Christensen A. M., Willemoes-larsen H., Lundby L., Jakobsen K. B. (1994). Postoperative throat complaints after tracheal intubation. *British Journal of Anaesthesia*.

[11] Hu B., Bao R., Wang X. (2013). The size of endotracheal tube and sore throat after surgery: a systematic review and meta-analysis. *PLoS ONE*.

[12] Metheny N. A., Clouse R. E., Chang Y.-H., Stewart B. J., Oliver D. A., Kollef M. H. (2006). Tracheobronchial aspiration of gastric contents in critically ill tube-fed patients: frequency, outcomes, and risk factors. *Critical Care Medicine*.

[13] Zanella A., Scaravilli V., Isgrò S. (2011). Fluid leakage across tracheal tube cuff, effect of different cuff material, shape, and positive expiratory pressure: a bench-top study. *Intensive Care Medicine*.

[14] Schumann R., Gandhi P., Switkowski K., Grant M. A., Bonney I. (2013). The volume and pH of residual pharyngeal fluid aspirated from the TaperGuard Evac endotracheal tube following elective surgery: a prospective pilot study. *Minerva Anestesiologica*.

[15] Tsuboi S., Miyashita T., Yamaguchi Y., Yamamoto Y., Sakamaki K., Goto T. (2013). The TaperGuard endotracheal tube intracuff pressure increase is less than that of the Hi-Lo tube during nitrous oxide exposure: a model trachea study. *Anesthesia and Analgesia*.

[16] Jordi Ritz E.-M., von Ungern-Sternberg B. S., Keller K., Frei F. J., Erb T. O. (2008). The impact of head position on the cuff and tube tip position of preformed oral tracheal tubes in young children. *Anaesthesia*.

[17] Asai T., Liu E. H., Matsumoto S. (2009). Use of the pentax-aws in 293 patients with difficult airways. *Anesthesiology*.

